# Beyond co-creation: why community-centered research ecosystems and advancing community research capacity building is implementation science’s next frontier

**DOI:** 10.1186/s43058-026-01032-8

**Published:** 2026-09-28

**Authors:** Ashley Hui Ying Teh, Shao Yuan Chong, Eleanor J. Ong

**Affiliations:** 1 Luskin School of Public Affairs, University of California, Los Angeles, Los Angeles, USA; 2 Department of Psychology, University of Denver, Denver, USA; 3 Saw Swee Hock School of Public Health, National University of Singapore, 12 Science Drive 2, #10-01, Singapore, 117549 Singapore

**Keywords:** Community-based participatory research, Implementation science, Capacity building, Health equity, Community engagement, Research sustainability, Citizen Science, Co-creation, Marginalized communities, Community-centered research ecosystems

## Abstract

Implementation science has increasingly embraced community engagement through co-creation and community-based participatory research, yet capacity for conducting implementation research itself remains concentrated within academic institutions. This commentary argues that current approaches perpetuate structural dependencies where communities require academic intermediaries to validate knowledge about their own contexts. While implementation science invests substantially in capacity development for practitioners and researchers through training institutes and mentorship programs, these opportunities systematically exclude the communities we claim to partner with. Existing frameworks position communities primarily as sources of contextual knowledge rather than independent implementation scientists, creating bottlenecks in addressing health inequities and limiting the sustainability of implementation research capacity. Drawing on case studies from *kayos kollective*, a Singapore-based community research group, we illustrate how communities can develop implementation research competencies through structured capacity building that integrates research training with advocacy and program development. These examples demonstrate pathways for community members to gain methodological skills, intellectual capital, and sustained research infrastructure beyond episodic consultation models. We propose shifting from community-based participation toward community-formed research ecosystems standing infrastructures that support communities in identifying implementation problems, adapting frameworks, collecting and analysing data, and conducting advocacy independently of external investigators. This reframing positions communities as producers of implementation science rather than periodic participants and redefines academic partners as capacity builders and technical consultants. Such ecosystems preserve methodological rigour while enabling locally driven innovation and ongoing implementation learning that current participatory models cannot support. Community-formed research ecosystems also address a long-standing gap in the field: most interventions persist beyond the lifespan of research partnerships, yet communities are rarely equipped to evaluate fidelity, identify emerging barriers, or make evidence-informed adaptations once academic partners disengage. By embedding analytic and evaluative capacity directly within communities, this model creates a mechanism for sustaining implementation learning, preserving context-specific knowledge, and expanding the field’s understanding of real-world implementation processes. Ultimately, decentralizing methodological authority in this way strengthens community research autonomy, supports equitable and durable implementation practices, and advances a more legitimate redistribution of research power.

Contributions to the literature • Demonstrates how community-formed research ecosystems can sustain implementation research capacity independently of academic oversight through case studies from Singapore.

• Proposes shifting from episodic consultation models to embedded research competencies that enable communities to conduct implementation research autonomously, furthering long term equity.

• Reframes academic partnerships from lead investigator roles to capacity builder functions, positioning communities as producers rather than participants in implementation science.

• Proposes the first articulated framework for a community-formed research ecosystem, advancing the field beyond project-based co-creation and CBPR models.

## Background

Implementation science has made significant strides in recognizing the importance of community engagement, with co-creation and community-based participatory research (CBPR) becoming increasingly prominent in the field [1]. This has narrowed the research-to-practice gap by centering local knowledge through shared decision-making, and the active involvement of communities throughout the research and implementation process. Despite becoming adept at involving communities in intervention design and adaptation, capacity is rarely transferred to conduct implementation research itself [2]. This perpetuates a gatekeeping system where communities remain dependent on academic researchers to translate their experiential knowledge into institutionally recognized forms.

Paradoxically, implementation science has invested substantially in capacity development for practitioners and early-mid career researchers through structured mentorship programs, training institutes, and professional development frameworks [3–5]. Yet this emphasis on capacity building conspicuously excludes the very communities we claim to partner with through co-creation. While we invest in teaching practitioners about implementations science frameworks such as Consolidated Framework for Implementation Research (CFIR), and Exploration, Preparation, Implementation, Sustainment (EPIS) and implementation strategies, communities, particularly marginalized populations bearing the greatest burden of health inequities, are left without equivalent opportunities to develop these capacities [6, 7]. Throughout this commentary, “community” refers to individuals with lived experience of the issues under investigation, including those without formal research training, as well as grassroots organizations and advocacy groups working in solidarity with affected populations. We recognize that community is not a uniform category, it encompasses considerable variation in organizational structure, research exposure, and proximity to academic institutions. The ecosystem model we propose is intended to be adaptable across this range.

Current implementation science frameworks like CFIR [8] and EPIS [9] emphasize context and engaging stakeholders. Yet these frameworks position communities primarily as sources of contextual knowledge or intervention recipients, rather than as independent implementation scientists in their own right. Even the most participatory approaches typically involve communities in identifying implementation barriers, adapting interventions, or collecting data, but the analysis, interpretation, and dissemination remains largely controlled by academic partners [10]. Implementation science expertise is thus reserved for professionals and academics, while communities remain subjects rather than scholars of their own implementation contexts.

This disparity has particularly problematic implications for marginalized populations, where implementation challenges are ongoing and context-specific [7]. While implementation science has extensively documented intervention sustainability, it has largely neglected the sustainability of implementation research capacity in communities [11]. While interventions may persist even after research partnerships end, communities are often left without the tools to assess systematically whether interventions continue to be implemented as intended, to identify emerging implementation barriers, or to make evidence-informed adaptations.

The lack of community research capacity creates bottlenecks in addressing health inequities. Implementation questions in under-resourced settings far exceed the capacity of academic researchers to address them [12]. Communities have intimate knowledge of their local implementation context, what Damschroder and colleagues (2009) describe as the “inner setting”, and often learn from and adapt implementation processes in real-time based on their experiential knowledge. However, without methodological tools to systematically document these adaptations and learnings, this rich implementation knowledge remains tacit, localised, and unavailable to inform broader implementation efforts [13]. Communities cannot assess fidelity to adapted interventions, measure implementation outcomes systematically, or contribute their context-specific insights to the implementation science literature unless they partner again with academic researchers, perpetuating cycles of dependency [2]. The result is a loss of valuable implementation knowledge and the perpetuation of the translation gap between evidence and practice in communities that could benefit most from implementation science.

## Where we are writing from: a short reflexivity statement

The commentary was developed by scholars as well as community members from *kayos kollective*, an Asian community group involving community researchers and members who are invested in the Asian LGBTQIA+ community welfare across a range of ages, genders, sexualities and education levels across diverse disciplines (e.g., psychology, public health, other natural and social sciences). In writing this commentary, we aim to speak to academic communities — particularly researchers who seek to address marginalised communities — with the goal of creating a blueprint for researchers and experts who may wish to resource communities to embark in self-sustainable community-based research. In doing so, this commentary aims to map for researchers and experts how best to empower marginalised communities with the skills to engage in rigorous, community-benefitting research, rather than being permanently subjected to reliance on guidance by researchers and academics.

We choose to write a reflexivity statement — one that highlights the ways in which we reflect on how our lived experiences impact our understanding of the topic — rather than a positionality statement. We make this decision in recognizing that the explicit naming of identities not only places some of our researchers and community members in danger given their vulnerabilities, but also in recognizing that the function of a positionality statement is for the purposes of acknowledging how lived experiences must necessarily shape our epistemological thinking surrounding a specific research topic. The lived experiences of being part of/supporting Asian LGBTQIA+ community members, and our investment in the community may create a stronger focus on ‘insider’s perspective’ and adopt a primarily grassroots perspective when offering our recommendations and suggestions. We use our lived experiences and/or our professional experiences working specifically with Asian LGBTQIA+ populations as a reference point, to consider ways in which community members can be better centered in the course of implementing research intended to serve marginalised communities.

Singapore’s sociopolitical landscape – including limited formal protections for LGBTQIA+ communities and a research environment historically concentrated within academic institutions – renders it a productive illustrative case for this argument. This commentary is not intended as a Singapore-specific analysis; rather, Singapore surfaces with particular clarity the conditions under which community-centered research ecosystems become not merely desirable but necessary: where affected communities cannot rely on institutional advocacy, grassroots knowledge production becomes a primary mechanism for ensuring their needs shape research agendas.

## Next steps: empowering through training for sustainable innovations for marginalized populations

How can we bridge this gap to begin building research capacity with the community, to ensure that impacts left by interventions can be sustained? We consider two case studies from *kayos kollective*, a Singaporean-based community research group focused on producing intersectional projects. *kayos* was first established with the commitment to empower marginalized community members to be equipped with the skills to do their own research and education work that can meaningfully advance the welfare of communities they care for. *kayos* primarily targets community members who would traditionally be excluded by the elite academic circle, with the intention of challenging the academic glass ceiling that keeps marginalized communities out of the field. Specifically, *kayos* drives research and education using the Socio-Scientific Progression by Leveraging on Intersectionality and Citizen-led Equity-driven (SPLICE) Research Framework, centering “Research Co-Creation,” “Safety For All,” “Reflexivity,” “Growth for All,” “Dynamic Ecological Context,” “Interlocking Systems of Oppression,” and a “Community-First Approach” [14]. The profile of *kayos* members range across educational experience and demographics, most of whom have little to no prior research experience.

In this commentary, case studies are deemed particularly relevant given its ability to capture context-dependent experiences. In this case, the importance of grounding community capacity building amongst marginalized communities calls for the need to employ context-rich evidence, rather than big-sample-size examples that erase context from discussion [15]. In *kayos’* case studies, mechanisms of research and program intentionally built in marginalized communities to contribute to the equity-oriented research and programs that *kayos* is running.

Having established the importance of citizen science involvement in implementation science research previously [16, 17], we focus on how a safe training space throughout each phase of research/program has permitted for community members to understand the mechanisms of equity-oriented research and programs, to later run their own research and programs as part of their capacity building. Importantly, there has not been any publicly-available “training program/curriculum” that allows for a neat adaptation to be used in *kayos* given the unique profile of the community research group; consequently, trainings were developed based on the needs of the members, the broader community as well as the projects that the team was working on (albeit always co-determined with the involved community members).

## Creating impacts beyond the LGBTQIA+ community: developing a simulated patient (SP) workshop to encourage inclusive medical practice for LGBTQIA+ individuals

The SP workshop first emerged as a community initiative: approached by *Project Prism*, a student group centering advancement of LGBTQIA+ interests within the medical student space, *kayos* initiated potentially conducting a mixed research-workshop that sought to understand medical students’ knowledge, attitudes and skills in terms of offering LGBTQIA+-affirming clinical care, specifically in relation to how they communicate with LGBTQIA+ community members. From the research design stage, *kayos* members were trained to conduct *surveys that employed LGBTQIA+-affirming framing*,* focus group discussions that centered discussions that mattered in determining LGBTQIA+-related issues*,* as well as ethnographic notetaking* to ensure that comprehensive data was collected. The unique positionality of *kayos* members as LGBTQIA+-focused community researchers ensured that interpretation of data as well as research execution consistently centered the LGBTQIA+-community’s welfare (e.g., when observing medical student participants’ reaction during various segments of the workshop). As the research design was formalised, *kayos* members were also trained to act as *community observers*,* script co-creators and Standardised Patients*, where community members were not only delivering parts of their own stories into the scripts to train medical students to ensure ecological validity, but also taking on the role of the actors and community observers to provide immediate feedback for improvement. Many of these skills would later translate to career advancement opportunities, as beyond the program itself, community members could request to join a longer list of trained SPs to keep doing the work while being paid for their service.

The study was meaningful in that community members picked up skills that would serve them beyond the study, but they also witnessed actual changes in approaches and improvement in skills amongst participants who joined the workshop, the very goal they sought to volunteer their time to achieve. The short-term expected outcomes were intended towards informing greater awareness vis-a-vis LGBTQIA+-affirming medical care, but the longer-term goals involved encouraging changes in the medical curricular in Singapore to better align with goals of promoting health equity through equitable medical care. Immediate survey feedback suggested that the shorter-term goal may have been achieved, but at this time of writing, formal integration and change in medical curricular remain unseen.

Despite the clear importance and relevance of the program in advancing the welfare of local LGBTQIA+ community members as well as the benefit that the funding could have brought about, grant applications for this study have been unsuccessful. With the continued opacity surrounding academic processes such as grant writing and acceptance, the lack of substantive feedback has translated to an inability for junior researchers situated within the community (rather than academic institutions) to improve their applications, compromising on the impact that they could otherwise create with financial resources backing the study. A more fundamental problem may be that dominant funding mechanisms were not designed with community-driven or citizen science research as a recognized mode of knowledge production. Major public funding bodies including national research councils and health research institutes typically require institutional affiliation as a condition of eligibility, effectively gatekeeping access to resources along lines that map onto academic credentialing rather than research relevance or community need. Opacity in the grant feedback process thus compounds this prior factor, meaning that ultimately, communities cannot improve applications to meet standards that were never designed with them in mind. This experience further reflects how academic standards remain imposed upon community-centered research, where “legitimacy” for funding eligibility are based upon divorced academic focuses, rather than what is truly meaningful and useful for marginalized communities of interest.

Ultimately, while the project was minimally funded by *Project Prism*, who had kindly offered a stipend to all stakeholders using their student club funding, the actual number of man hours invested to design the program, train community members, implement the workshop and analysed the research data collected made it such that the stipend could not amount to a comparable salary for community members; instead, community members had to simply take the experience as one that was ‘voluntary’ rather than appropriately reimbursed. In this sense, the burden of making the program happen landed on the community members — offering unpaid labour to realizing the program.

## From evidence to action: community-driven submission of comments for revision of the American Psychological Association’s 2017 Multicultural Guidelines

One of the selected projects in which community members initiated was contributing to the Revision of the American Psychological Association (APA)’s 2017 Multicultural Guidelines, of which APA was calling for public comments on. The APA Multicultural Guidelines was initially developed to guide psychology researchers and clinicians to better account for multiculturalism within their research and practice. The current call for inputs for revision is from APA itself, recognising that it has been about 7 years since the implementation of the guideline. While situated in the U.S., given the global influence of APA in informing psychological research and practice beyond American borders, *kayos* members articulated a need to contribute to this call for revision to offer a more global, minoritised community-centered perspective, especially recognising the impact the APA has had on Singaporean research and practice, where an equivalent guideline has not been published for the local psychology community.

Recognising the global influence of the APA, *kayos* members felt compelled to call upon the APA to de-center the Euroamerican-centric lens even its Multicultural Guidelines maintained, and to also further emphasize on the need to center intersectionality in its full complexity. Through this advocacy exercise, informal hands-on mentorship training involved *(1) Scaffolding and modelling critical engagement with target material for advocacy* (in this case, the Multicultural Guidelines); *(2) Brainstorming to center what the community members saw as priority based on lived experiences* (decentering the researchers’ academic instincts); *(3) Guided advocacy-focused research and writing* (writing together rather than individually; thinking about selecting scholarship that centered the advancement of epistemology that are social-justice oriented where possible). Aside from allowing for the community-voice to be amplified, the end report centered on ways in which the discipline in research, practice and community engagement can meaningfully advance justice from a global lens. Community members who were engaged also took away skills and knowledge that informed their later advocacy works within and outside of *kayos.*

## Moving past community-based: towards community-centered research ecosystems

Drawing on these lessons from *kayos*’s previous work, where community members were trained in research methodologies, co-designed research and then used these skills as methods to build their own intellectual and social capital, we propose a reframing of the dominant narratives in implementation science. The prevailing logic frames communities as research sites (locations where research happens rather than infrastructures from which research is generated) where a “gap” must be bridged. We believe that this framing limits imagined possibility. Even in CBPR and co-creation frameworks, communities are invited into researcher-defined spaces that often do not carry the explicit purpose of sustaining an independent community research infrastructure. While CBPR seeks to equitably involve community members, researchers and other stakeholders in all stages of the research to produce positive, transformative and sustainable change with, for and in community, emphasizing [18, 19] long-term partnerships, shared decision-making, reflexivity and capacity-building, as an innovative research paradigm that combines knowledge and action to improve community health and reduce disparities [10, 19], the research infrastructure itself usually stays with universities or service organizations. Rather than simply aiming to close the gap, we argue for bringing research competencies directly into the community in a sustainable way, and examining what new methodologies, analytic lenses and implementation practices can emerge.

**Fig. 1:**
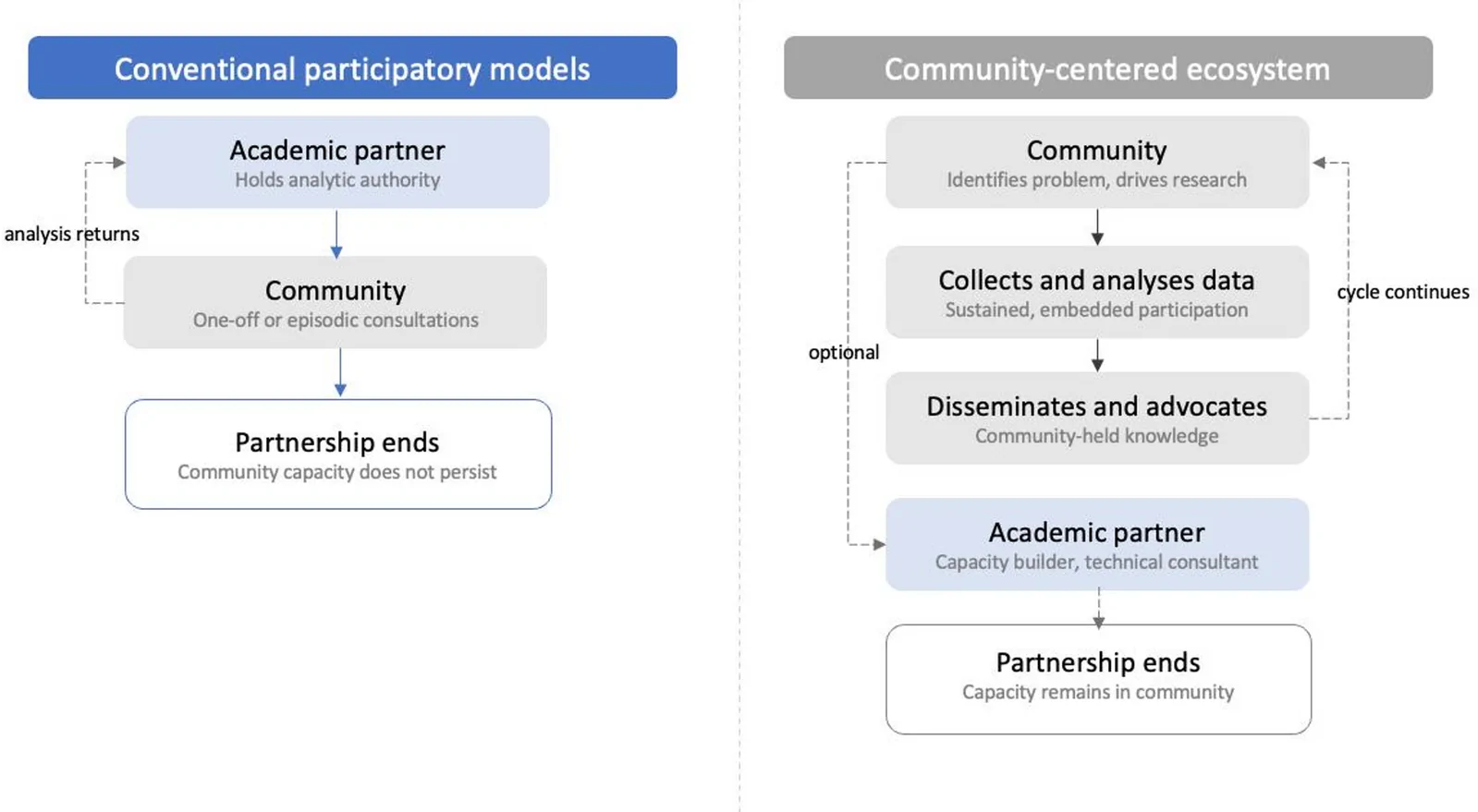
Conventional participatory models vs. community-centered research ecosystem: a structural comparison

This would involve shifting from the typical model of project-based participation and consultation, where communities are convened without being truly embedded in the ongoing analytic and interpretive work. One way to do this, we propose, is to build a community-centered research ecosystem: a standing, community-based infrastructure that enables members to carry out implementation research both with and independently of external investigators and academics, differentiated from conventional participatory models (Fig. 1).

### What is a community-centered research ecosystem?

A community-centered research ecosystem is a way of *building* and *sustaining* a cohort of community researchers who can:

1. Identify implementation problems relevant to their context.
2. Select and adapt appropriate implementation frameworks and theories.
3. Collects and analyzes data on implementation processes and outcomes.
4. Document and research adaptations and operational learning as they conduct research.
5. Conduct advocacy or other programmes informed by their findings.
6. Sustain and evaluate interventions that persist beyond initial research partnerships.
7. Establish continuity in capacity-building by disseminating knowledge to their own communities and networks, and by training subsequent members who join the ecosystem.

### Practical insights for building a community-centered research ecosystem

To operationalize these ecosystems, training must move beyond academic theory toward a “safe training space” that prioritizes community welfare and ecological validity. Based on the kayos kollective experience, effective training involves the following actionable elements:

1. Scaffolding and Modeling: Using hands-on mentorship to model critical engagement with materials, such as clinical guidelines or policy documents, to prepare members for independent advocacy.
2. Integrated Competency Building: Integrating research training directly with program delivery, such as training community members as simulated patients or observers who provide immediate feedback to ensure ecological validity.
3. Community-Led Priority Setting: Centering what community members identify as priorities based on lived experience rather than purely academic instincts.
4. Collaborative Production: Employing “guided research and writing” where teams write together with intentionally selected scholarship in alignment with priorities set by the community.
5. Self-Sustaining Knowledge Infrastructure: Formalizing independent publication and co-authorship pipelines to build a community-led publication record, ensuring continuity in capacity-building by using generated knowledge to train subsequent ecosystem members.

We acknowledge that community-led monitoring bodies, advocacy coalitions, and community-led clinics represent important existing mechanisms with overlapping functions. What distinguishes a community-centered research ecosystem is the intentional embedding of implementation science competencies including systematic data collection, framework adaptation, and evidence synthesis, within community infrastructure, enabling communities to generate and interpret implementation evidence rather than solely acting on it. Resources enabling this include sustained investment in training, co-authorship opportunities, and funding structures that do not require academic institutional backing for eligibility. This ecosystem would eventually become more self-sustaining, built on repeated cycles of training, joint research, and opportunities within and without individual research projects. The case studies mentioned already demonstrate successful elements of this, where community members learn research methods and later apply their skills into policy-facing advocacy.

We propose this model not only as a practical strategy for strengthening and furthering the field, but as an argument for the recognition and legitimisation of community-centered research ecosystems. A community-centered research ecosystem creates pathways for community members to build intellectual, social and professional capital that are rarely accessible under conventional CBPR or co-creation models. Co-authorship with community partners strengthens engagement, improves implementation relevance and deepens capacity [20]. An ecosystem approach makes these opportunities the standard rather than as something episodic advancing a paradigm where community researchers hold standing as producers of implementation knowledge. Yet we would be remiss not to acknowledge that funding access is not purely a function of capacity or credibility. Funding landscapes are shaped by political arrangements and institutionally constructed definitions of what counts as rigorous, legitimate, and fundable research — definitions that have historically centred academic infrastructure as a proxy for quality. Community-centered research ecosystems do not resolve this by making communities more competitive within existing arrangements alone; they create a basis from which communities can name, contest, and seek to reshape these arrangements. Sustainable investment in community research capacity therefore requires not only better-resourced communities, but funders willing to interrogate the political assumptions embedded in their own eligibility criteria.We do not claim that communities unilaterally define research agendas and can never involve others, but rather that the distinction lies in intention. We argue for embedding research competencies directly within communities in a way that enables them to generate, interpret, and act on implementation evidence without continual academic oversight. The question is not whether academic and community partners can collaborate well, but whether communities retain analytic and evaluative capacity when those partnerships end. Improved collaboration models do not resolve this gap, only the transfer of methodological infrastructure does. In such ecosystems, new analytic perspectives, methodological adaptations, and implementation strategies can emerge from lived experience and local epistemologies. This shifts the conversation away from episodic consultation and support and toward sustained investment in community researchers as an integral part of the field’s capacity.

## Conclusion

This reframing has practical consequences. Ultimately, community-centered research ecosystems rest on a foundational reorientation: research as a means through which communities surface and address their own needs, rather than as a mechanism through which external investigators determine what those needs are and how they should be met. It alters the role of the academic partner from lead investigator to capacity builder and technical consultant. It also changes what counts as a successful collaboration: the goal is not only a completed project, but a functioning research ecosystem capable of sustaining ongoing implementation learning. By grounding methodological authority within the community, this model positions communities as producers of implementation science rather than periodic participants in it. This is a way for implementation science to move beyond community-based involvement and toward community-formed research capability in a way that preserves rigour, addresses persistent concerns about sustainability, and opens space for locally driven innovation that project-bound and indeed participatory models currently cannot support.

## Data Availability

No datasets were generated or analysed during the current study.

## Abbreviations

APA: American Psychological Association
CBPR: Community-Based Participatory Research
CFIR: Consolidated Framework for Implementation Research
EPIS: Exploration, Preparation, Implementation, Sustainment
LGBTQIA+: Lesbian, Gay, Bisexual, Transgender, Queer/Questioning, Intersex, Asexual, and others
SP: Simulated Patient
SPLICE: Socio-Scientific Progression by Leveraging on Intersectionality and Citizen-led Equity-driven
